# Understanding physical drivers of the 2015/16 marine heatwaves in the Northwest Atlantic

**DOI:** 10.1038/s41598-021-97012-0

**Published:** 2021-09-02

**Authors:** E. Perez, S. Ryan, M. Andres, G. Gawarkiewicz, C. C. Ummenhofer, J. Bane, S. Haines

**Affiliations:** 1 Department of Physical Oceanography, Woods Hole Oceanographic Institution, Woods Hole, MA 02543 USA; 2 Department of Marine Sciences, University of North Carolina-Chapel Hill, Chapel Hill, NC 27599 USA

**Keywords:** Physical oceanography, Climate-change impacts

## Abstract

The Northwest Atlantic, which has exhibited evidence of accelerated warming compared to the global ocean, also experienced several notable marine heatwaves (MHWs) over the last decade. We analyze spatiotemporal patterns of surface and subsurface temperature structure across the Northwest Atlantic continental shelf and slope to assess the influences of atmospheric and oceanic processes on ocean temperatures. Here we focus on MHWs from 2015/16 and examine their physical drivers using observational and reanalysis products. We find that a combination of jet stream latitudinal position and ocean advection, mainly due to warm core rings shed by the Gulf Stream, plays a role in MHW development. While both atmospheric and oceanic drivers can lead to MHWs they have different temperature signatures with each affecting the vertical structure differently and horizontal spatial patterns of a MHW. Northwest Atlantic MHWs have significant socio-economic impacts and affect commercially important species such as squid and lobster.

## Introduction

**Figure 1:**
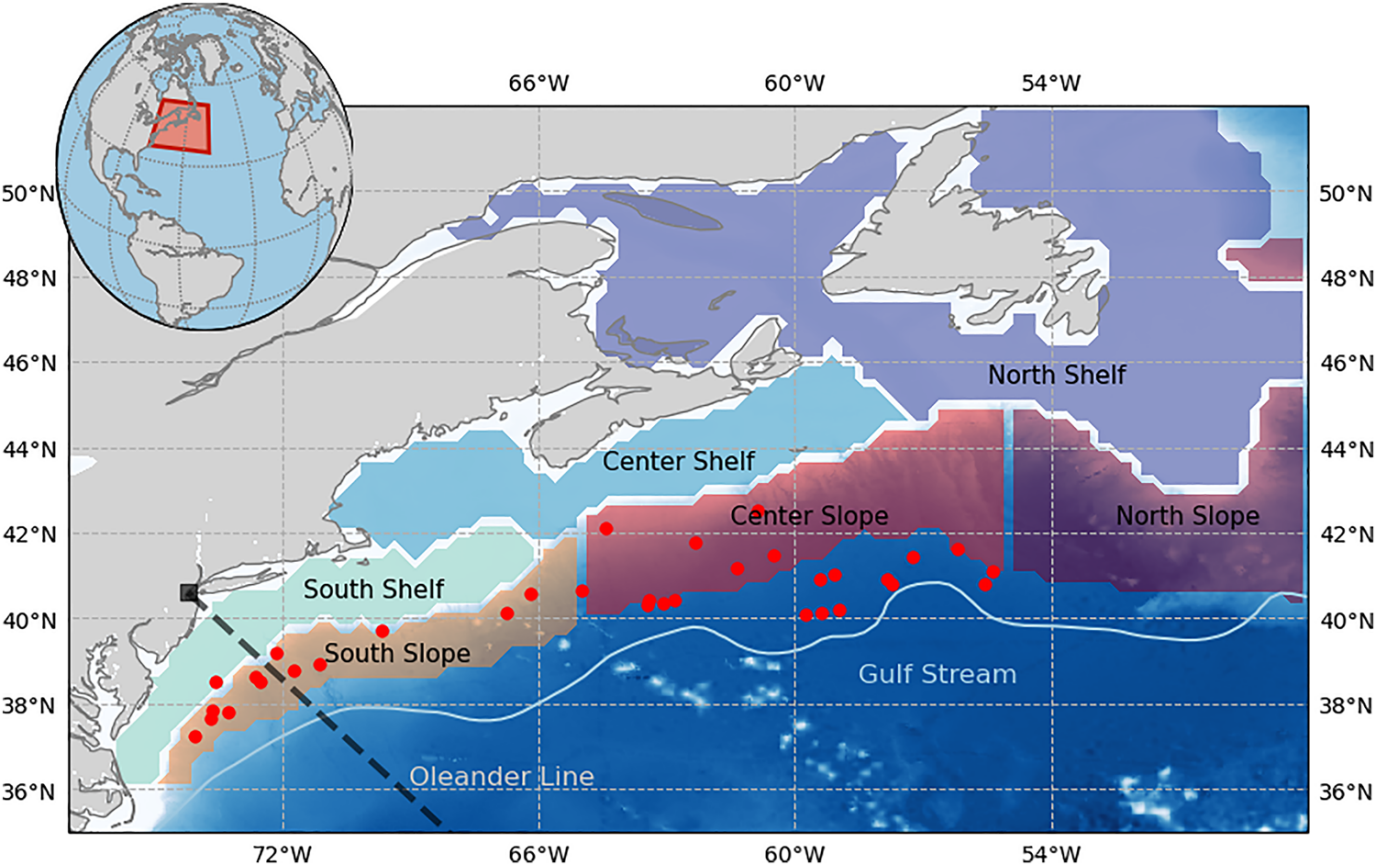
Map of the Northwest Atlantic. Positions of warm core rings (WCRs) on their date of absorption for the 15-month period considered here are indicated with red dots. The dashed (black) line indicates the Oleander line. The light blue line is the average path of the Gulf Stream (1993–2017). Shaded boxes are sub-regions used for marine heatwave analysis. This map was generated using Python version 2.7.5.

Marine heatwaves (MHWs), defined as discrete events of anomalously warm ocean temperatures, can disrupt marine ecosystems and hence human industries like fisheries, tourism, and aquaculture. MHWs can occur throughout the global ocean and across seasons. Under anthropogenic climate change, more intense and frequent MHWs are expected^1,2^. The Northwest Atlantic (NWA, see Fig. 1), where waters are warming four times faster than the global average, has experienced notable MHWs in the last decade^3–6^. This region is home to a highly productive ecosystem with significant economic importance, and includes the highest-valued seafood port in the United States, located in New Bedford, MA^7^. Recent MHWs in 2012 and 2016 have been among the strongest on record in the NWA^3,5,8^. In the NWA, 2012 was not only one of the warmest years on record, but also one of the most disruptive years to marine ecosystems and fisheries^5^. Given the economic implications and strong background warming in the NWA region, it is particularly important to understand drivers of MHWs, especially the relative role of oceanic versus atmospheric contributions.

Chen et al.^4,8^ showed that a MHW in 2012 in the NWA was widespread along the continental shelf, reaching from Cape Hatteras to the Gulf of Maine. In 2012, the sea surface temperature (SST) from Cape Hatteras to the Grand Banks was 2 °C higher than the 1982–2011 average^5^. This warming coincided with an unusually northward-shifted atmospheric jet stream position that persisted from the end of 2011 into 2012 and confined the polar air masses to its north. Warm southerly air remained over the northeast U.S. and neighboring continental shelves for much of the winter, which altered air-sea heat fluxes and reduced heat loss from the oceans, and caused the 2012 MHW^4,8^.

Recent variability of the Gulf Stream has likely also contributed to warming of the continental shelf and slope in this region^6^. Gulf Stream meanders form further to the west than in the 1990s, and are of larger amplitude^9^. A regime shift in 2000 is associated with a substantial increase in the number of WCRs formed annually from Gulf Stream meander crests^10^. Large amplitude meanders and WCRs can bring warm salty water into close proximity to the continental slope and shelf break^11,12^ and can affect both the Shelfbreak Jet as well as shelf temperature^13^. Gawarkiewicz et al.^14^ found that an ocean-driven MHW along the continental shelf of the Mid-Atlantic Bight in 2017 was forced by the presence of a WCR, which resulted in cross-shelf transport of warm and salty Gulf Stream water. Additionally, a northward shift of the Gulf Stream toward the Tail of Grand Banks, where it potentially blocks the equatorward-flowing Labrador Current has been shown to increase salinity and temperature of subsurface waters on the Northwest Atlantic Shelf, “downstream” (southwest) of this interaction location^15,16^. Warmer, saltier waters southwest of the Tail of the Grand Banks may even reinforce atmospherically-initiated MHWs, such as the one in 2012 in the NWA^15^. Thus, both the jet stream motions in the atmosphere and Gulf Stream variability and rings in the ocean may serve as potential drivers for MHWs in the NWA.

In 2016, SST anomalies similar to those in 2012 were observed in the Gulf of Maine, which led to early lobster landings and a high total number of landings also in 2016^5^. While this had wide-reaching effects on the lobster fishery supply^5,17^, the drivers of the anomalously warm shelf temperatures have not been well studied to date. Additionally, MHW events can exacerbate negative impacts of the anthropogenic background warming that many iconic Northeast Shelf species like Atlantic Sea Scallop, Atlantic Cod, and Atlantic Mackerel are projected to experience^1,18^. Other economically important species, such as Inshore Longfin Squid and Butterfish, may potentially benefit from climate change^18^, and how they respond to MHWs is not well known.

In this study, we examine the seasonal evolution and spatial patterns of SST as well as temperature at depth along an expendable bathythermograph (XBT) repeat section to characterize MHWs detected in 2015/16. We investigate driving processes with a qualitative assessment of the relative roles of atmospheric forcing (by comparing the temperature patterns with jet stream latitude) and oceanic advection (by examining Gulf Stream position and meandering/ring formation).

The present study is organized as follows. Data and methods are described in “Data and methods” section. “Temporal evolution of the 2015/16 MHWs” section includes an analysis of MHWs detected in the region from October 2015 through 2016. “The relative role of possible MHW drivers” section investigates the role of the jet stream position, as well as Gulf Stream meandering, ring formation, and latent heat flux as possible drivers of these SST anomalies. The results are discussed in “Discussion” section.

## Data and methods

MHWs are identified as anomalously warm, prolonged events with SST anomalies exceeding the 90th percentile for a duration of 5 days or longer. This standard for defining MHWs using daily SST data^19^ was developed to allow comparison of different events across seasons and geographic locations. Hobday et al.^20^ built upon this MHW definition, proposing MHW categories based on intensity, which is the strength of the SST anomaly. We apply the MHW detection algorithm by Hobday et al.^19^, on area-averaged daily SST data (described below) over six sub-regions covering the shelf and slope region (Fig. 1) using a 30-year baseline from 1986 to 2016. The six sub-regions are amalgamations of the Shelf/Slope boxes defined in Chen et al.^6^, and are defined by combinations of their boxes (A–H and 1–11) as follows: our “South Slope” contains boxes A–B, “Center Slope” contains C–D, “North Slope” contains E–H, “South Shelf” contains 1–3 in the Middle Atlantic Bight, “Center Shelf” contains 4–7 corresponding to the Gulf of Maine and Scotian Shelf, and “North Shelf” contains 8–11 which is the Gulf of Saint Lawrence merged with the Newfoundland Shelf. Seaward edges of the Slope boxes are chosen to be roughly parallel to the main axis of the Gulf Stream to contain part of the Slope Sea, which is the region between the continental shelf and Gulf Stream.

The SST data used is the NOAA ¼° daily Optimum Interpolation Sea Surface Temperature (OISST)^21^ dataset comprising global SST data from 1982 to 2019. OISST blends observations from satellites, ships, buoys, and Argo floats^21^. Due to the long timescales over which the drivers of the MHW events operate, our other analyses (e.g., of water column temperature anomalies and jet stream position) are conducted with monthly-averaged data.

A Jet Stream Visualization (jsviz) tool^22^ provides maps and vertical sections of atmospheric pressure and high-wind features every 6 h based on the hourly 4D ERA5 atmospheric reanalysis data from the European Centre for Medium-Range Weather Forecasts^23^. The jet stream’s latitudinal position for a given longitudinal slice, is characterized by a maximum wind speed (greater than 40 m/s) within the upper-troposphere/lower-stratosphere. A 30-day low-pass Butterworth filter is applied to produce the jet stream’s latitudinal positions across 77–50° W. This time series of jet stream position is used here to examine the variability of the jet stream during 2015/16 and to compare this to other years. The jet stream latitudinal position climatology is based on the 30-year period 1986–2016. Due to the strong gradient in atmospheric temperatures that is associated with the jet stream, we interpret the position of the jet stream as a proxy for more or less heat flux into the ocean. Hence, an anomalously far south jet stream position would generally reduce the heat flux into the ocean to the north of the jet. While an increased heat flux would be associated with an anomalously far north jet stream position. To investigate heat fluxes, latent heat flux data from the JRA-55 atmospheric reanalysis product^24^ is used, which spans 1958–present. We focus on latent heat flux as Schlegel et al.^25^ found this to be the most relevant term that modulated the onset and decline of marine heatwaves in the NWA.

A time-mean Gulf Stream path is defined by the 25-cm sea surface height (SSH) contour in the 1993–2017 time-averaged SSH field calculated from daily satellite altimetry maps, as described in Andres^9^. The 25-cm SSH contour from monthly-mean SSH maps is used to investigate the time-variable Gulf Stream path, its proximity to the continental shelf, and to identify meanders. Additionally, to examine the roles of WCRs, which can be generated from pinched off meander crests^26^, the Warm Core Ring Census (1980–2017) as described in Gangopadhyay et al.^27,28^ is used to establish the interannual variability in the number of WCRs formed and to examine where the WCRs dissipated in 2015/16^27^.

Since 1977, XBTs have been deployed from the container ship CMV *Oleander* on its repeat track from Elizabeth, New Jersey to Hamilton, Bermuda^29^, providing a unique long-term dataset. To investigate the sub-surface expression (vertical structure of water temperature) of the MHW on the southern shelf and slope, we use the monthly XBT gridded temperature sections from the Oleander Line, described in Forsyth et al.^13^. These transects span the New Jersey shelf to the northern edge of the Gulf Stream from 1977 to 2018, and temperature anomalies relative to a monthly climatology are reported on a 10-km (cross-shelf) by 5 m (vertical) grid^30^. The cross-shelf positions are defined by the distance between the temperature profile and the 80-m isobath, which is the average location of the foot of the shelf break front off New Jersey^29,31^.

## Results

### Temporal evolution of the 2015/16 MHWs

**Figure 2:**
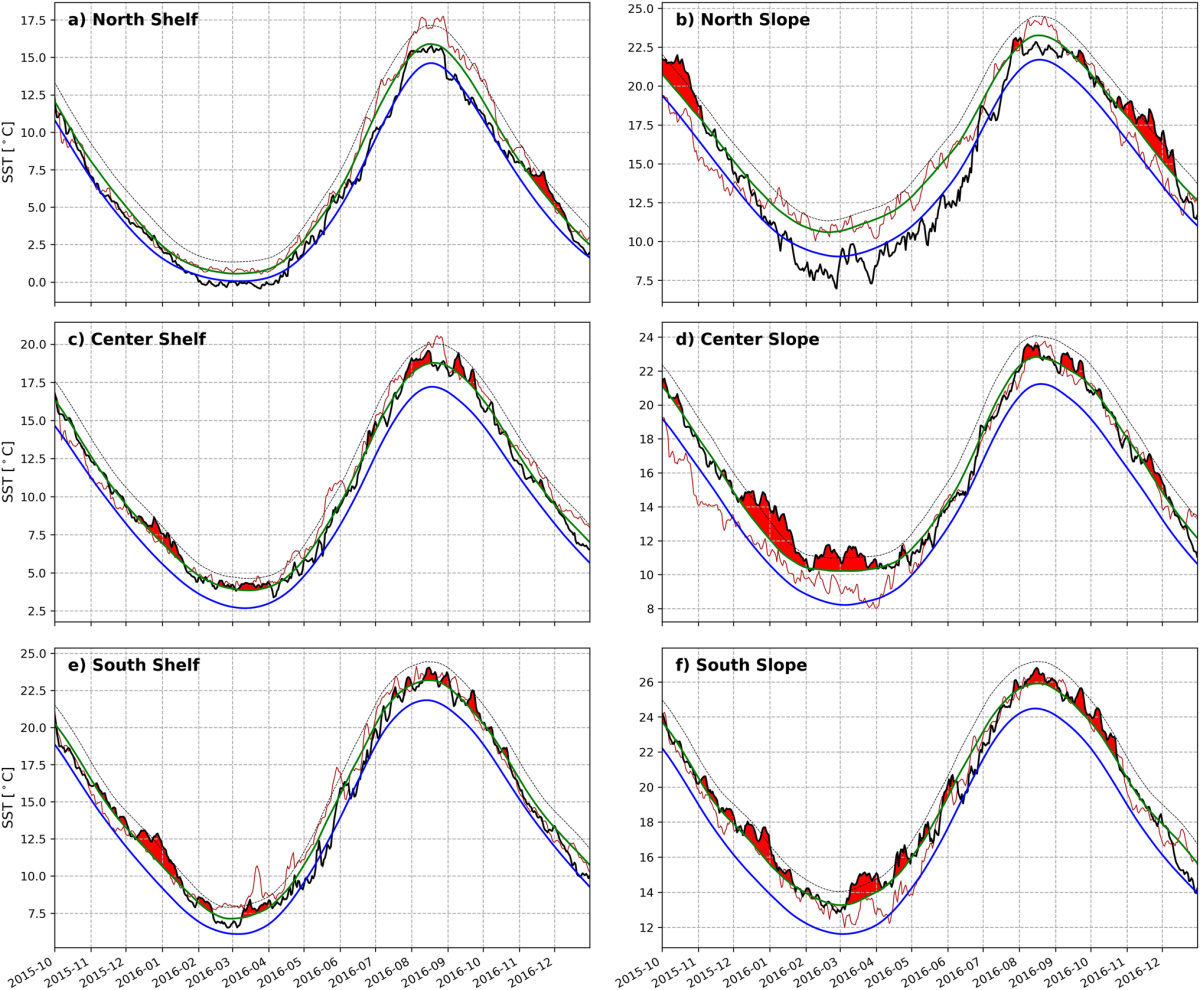
Evolution of the daily SST within sub-regions for October 2015 to December 2016 (black lines). The October 2011 to December 2012 SSTs are shown for comparison (red line). The blue line is the climatology based on a 30-year period (1986–2016). The green line is the threshold (90th percentile), as described in Hobday et al.^19,20^. The dashed line is twice the threshold, which categorizes a ‘strong’ MHW category. The red shading indicates a MHW. Note that the y-axes for all plots are different to increase visibility, since SST varies greatly from north to south.

We identify and examine MHWs in the NWA for the period October 2015 to December 2016. According to the surface MHW detection algorithm, there are a total of 7 MHWs for the entire NWA during this period, ranging in intensity from “moderate” to “strong”, and ranging in length from 5 to 42 days. Even though much of the NWA had elevated SST during late 2015 and 2016 (not shown), the NWA did not meet the MHW threshold continuously for the entire period. Rather, SSTs in the NWA flipped into (and then out of) MHW states, likely driven by various mechanisms throughout the year. We focus primarily on three periods in the 15-month period, which are the fall 2015, the winter 2015/16, and late summer 2016 into fall 2016, when multiple of the sub-regions experienced MHW conditions. The intensity of the MHWs varied geographically within the NWA shelf and slope region (Fig. 2). Each sub-region of the shelf and slope hence experienced “moderate” to “strong” MHWs, though not necessarily concurrently. The greatest regional SST anomalies were observed during winter 2015/16, with peak anomalies occurring in the South and Center Slope regions in January. By the end of November 2015, the northern regions had cooled (eventually even exhibiting negative temperature anomalies), but the center and southern sub-regions on the shelf and slope maintained elevated SST anomalies throughout the winter and early spring. In August, the anomalous warming became more widespread in all regions, and was especially intense in the Center/South Shelf and Slope. These spatial differences in the evolutions of SST anomalies averaged by sub-region, suggest that the MHWs in this time period were likely driven by different forcing mechanisms, discussed in “Discussion” section.

During the 15-month period, the entire NWA experienced 7 MHWs. Breaking it down further the combined Shelf (area-averaged over North, Center, and South) region experienced 4 MHWs and the combined Slope region experienced 8 MHWs. The longest and most intense MHWs were concentrated in the center and southern regions (Fig. 2). The South Slope experienced the most MHWs with a total of 13 events and had 248 MHW days (out of 457 days for the 15-month period). The region that experienced the least anomalous warmth was the North Shelf which experienced 2 MHWs with a total of 40 MHW days. The Center Shelf had 10 MHWs with a total of 166 MHW days. A 45-day long “moderate” MHW that lasted from February to April 2016 was the largest event of the ten. The South Shelf experienced 12 MHWs with a total of 163 MHW days. Its biggest event was a 40-day long “strong” MHW that started in December 2015 and ended in January 2016.

#### Fall 2015

**Figure 3:**
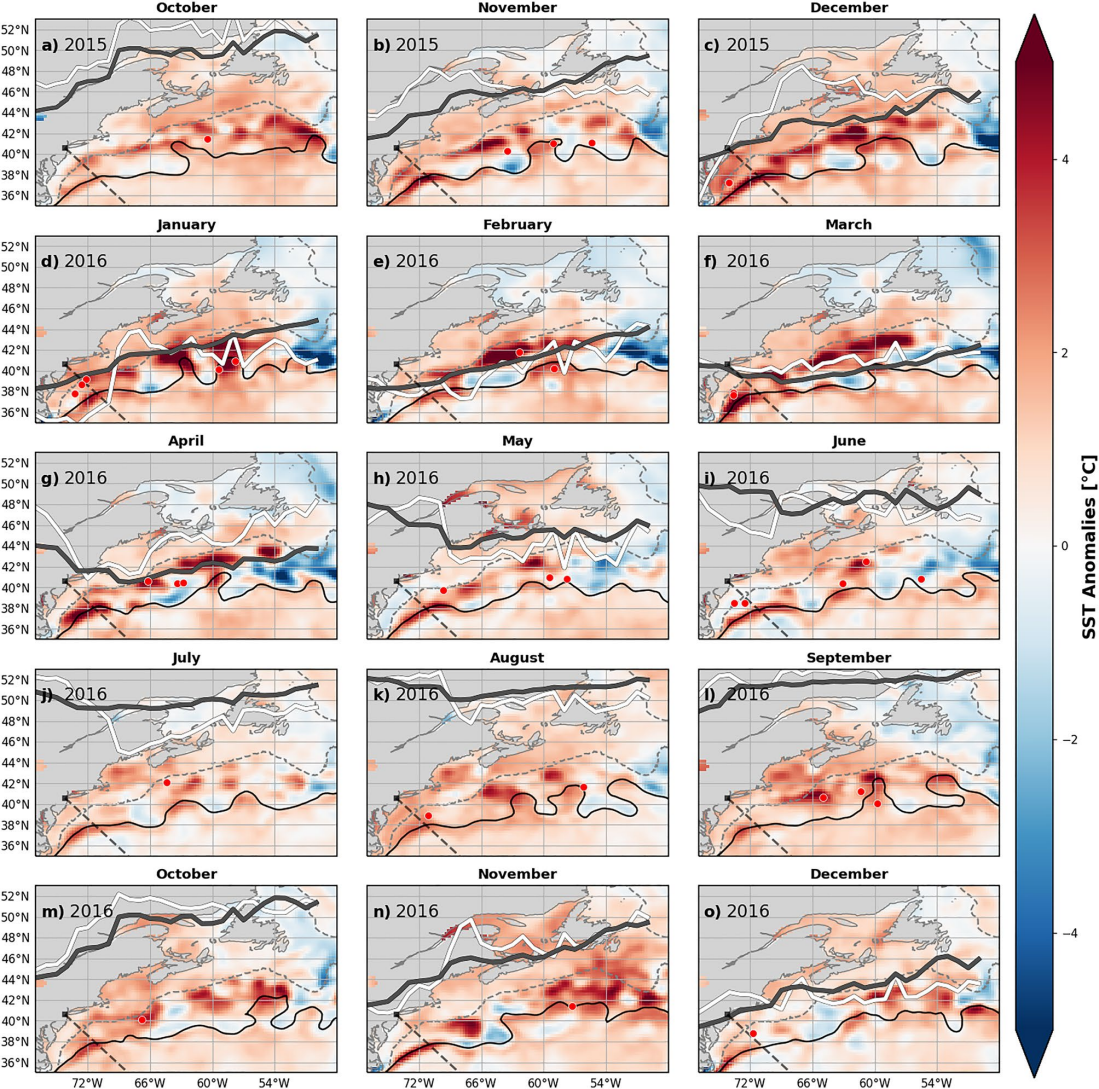
SST anomalies in the NWA for the 15-month period 2015/16. Black dashed line indicates the Oleander Line. Black solid curve indicates the average Gulf Stream path (based on 25 cm SSH isoline) for each month. Positions of WCRs on their date of absorption for the 15-month period are indicated with red dots. Gray dashed line is the isobath contour at 500 m. The thick gray line is the climatological position of the jet stream (1986–2016). The thick white line is the jet stream’s latitudinal position for 2015/16. These maps were generated using Python version 2.7.5.

**Figure 4:**
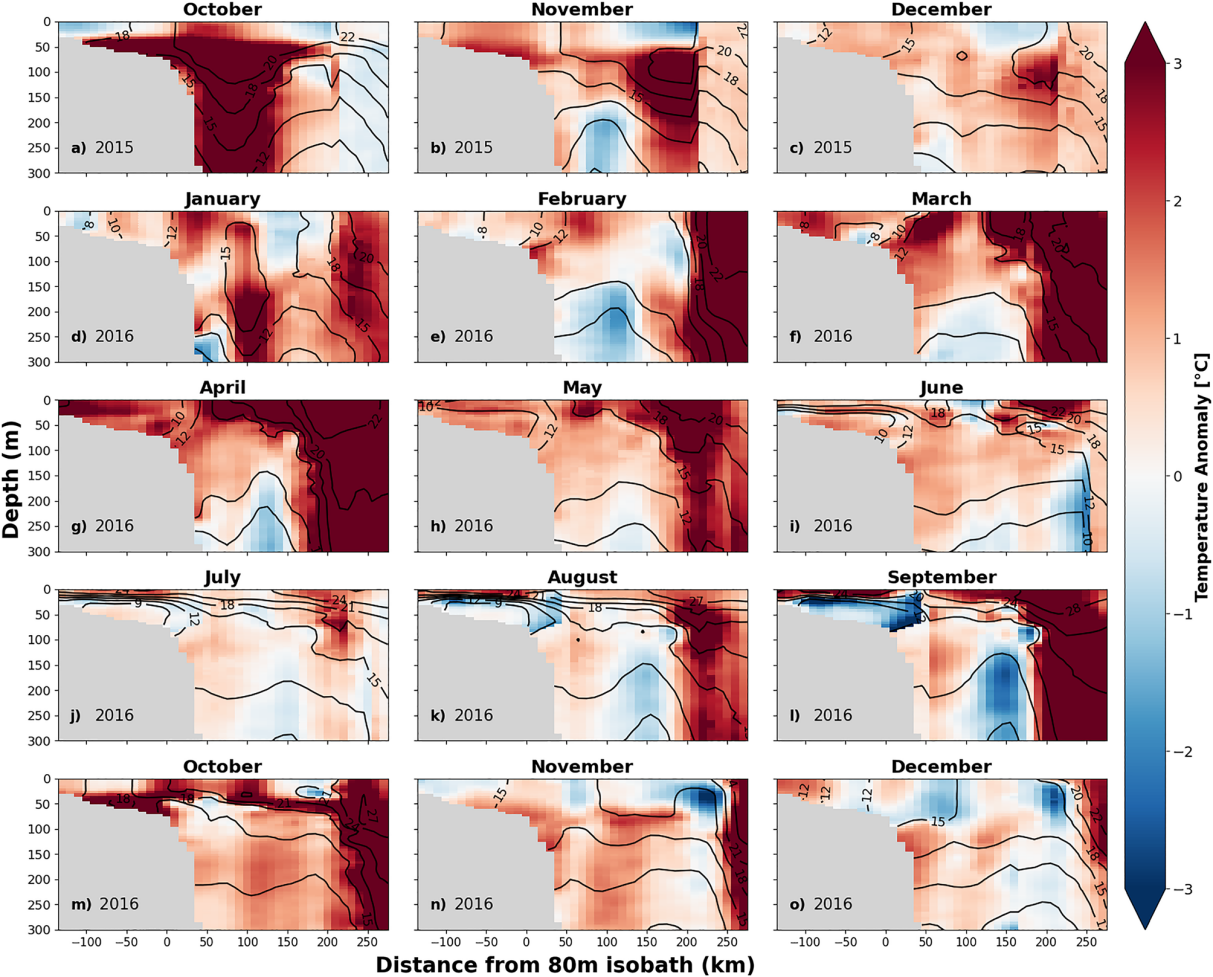
Vertical sections of temperature anomalies along the Oleander Line. Color shading indicates the temperature anomaly and the black lines indicate the isotherms. This figure was generated using Python version 2.7.5.

In October 2015, positive SST anomalies were widespread across the NWA (Fig. 3a). In the North Slope, South Shelf, and South Slope (Fig. 2b,e,f) “strong” MHW conditions were abating or had just ended. The Southern Slope had just experienced its largest event on record from February 2015 to October 2015, a 221-day long “strong” MHW. In the northern regions temperatures had begun to decrease. By November 2015, all other regions were still anomalously warm, though technically no longer in MHW states. During this same time, the jet stream was anomalously far north across most of the region 77–50° W, relative to the 1986–2016 climatology (Fig. 3a–c, white line). The jet stream latitudinal anomaly was especially pronounced in December 2015 over about 70–60° W, and was displaced northward by about 8° from its climatological position (Fig. 3c, white line). To get a sense of subsurface anomalies in the Slope and encroaching onto the Shelf, we consider the data from the Oleander line. These show subsurface temperature anomalies of > 3 °C, as well as anomalies of > 2 °C in the upper 50 m in October 2015 (Fig. 4a). By the end of fall 2015 and beginning of winter 2016, the strongest SST signals were seen in the Center and South Slope (Fig. 3b–d).

#### Winter 2015/16

As noted earlier, by the end of 2015 a MHW in the North Slope ended. Area-averaged SST anomalies in the northern regions became less pronounced and decreased to about a 0.5 °C anomaly in the North Shelf as the North Slope experienced a cold anomaly of −0.5 °C in December 2015. Despite this, SST anomalies in the center and southern sub-regions remained positive in December 2015, with area-averaged SSTs here exceeding the baseline by more than 1.6–2.6 °C. During this period, the uniform spatial expression of SST anomalies across the NWA, noted during October 2015 (Fig. 3a), changed over the course of December 2015 to April 2016 (Fig. 3c–g) as the SST anomalies became concentrated and intensified in the Center and Southern Slope. Additionally, along the Oleander Line in October and November strong temperature anomalies of > 3 °C are seen in Fig. 4a,b.

**Figure 5:**
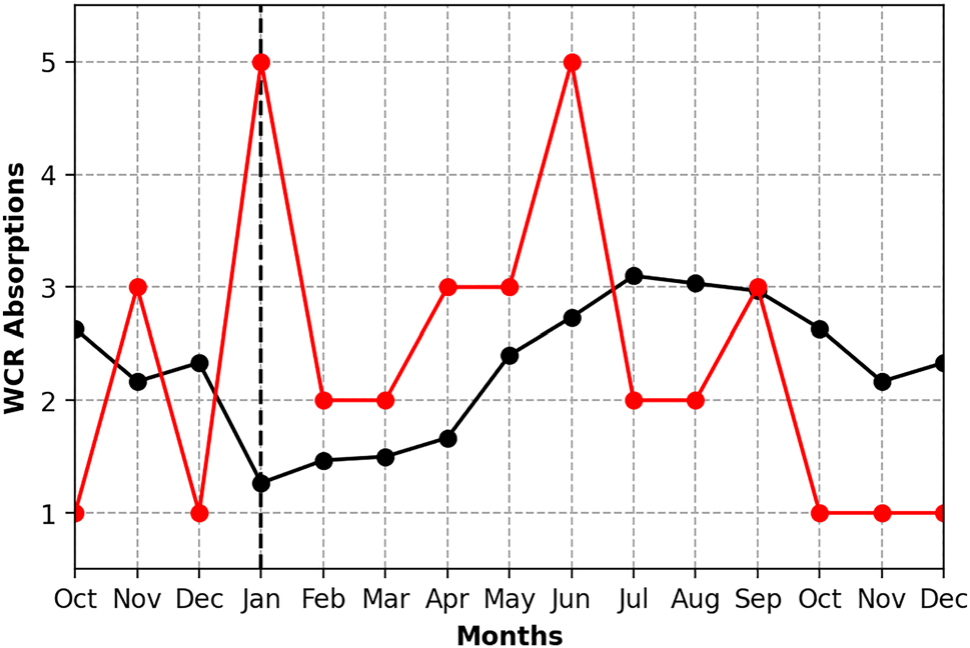
The number of WCR absorptions in the NWA from October 2015 to December 2016 (red line) is plotted against the absorption climatology for 1986–2016 (black line). An “absorption” occurs when a WCR merges back into the Gulf Stream or dissipates locally.

In concert with these SST signatures, large amplitude meanders of the Gulf Stream’s path (determined from the 25-cm SSH contour and denoted with the black curves in Fig. 3) evolved south of Nova Scotia and the Gulf of St. Lawrence from October 2015 to January 2016. Some of those meanders pinched off and formed WCRs, which tend to propagate southwestward along the continental slope. Notably, 7 WCRs were formed in the Center Slope in December 2015 (not shown), which is about 5 more than the mean (1986–2016). Climatologically, WCR formations are highest in summer and lowest in winter. July has the most, with about 3 WCRs formations on average. December has the fewest, with about 1 WCR formation on average^10^. Interestingly, 3 of those 7 WCRs formed in December 2015 were absorbed back into the Center and South Slope in early January 2016 (Fig. 3d, red dots). In total, 5 WCRs were absorbed into the Center and South Slope in January, which is about 4 more WCRs than usual (Fig. 5) and therefore likely contributing to the observed temperature anomalies.

The sub-region that experienced the most intense MHW during the winter 2015/16 was the Center Slope with a “strong” MHW that lasted 59 days, from December to January, with a mean SST of 13.4 °C (2.8 °C anomaly). The South Slope also experienced a “strong” MHW that lasted 44 days around the same time period, with a mean SST of 16.4 °C (2.4 °C anomaly). Concurrently the Center and South Shelf experienced MHWs, although the Center Shelf only experienced a “moderate” MHW. The South Shelf also experienced a “strong” heatwave that lasted 40 days from December to January and had an area-averaged SST of 11.9 °C (2.6 °C anomaly). This implies the impact of the warm slope waters on the continental shelf temperatures. Besides the anomalous position of the jet stream in December 2015, there are not many other anomalies/variability of the jet stream for January to March 2016 (Fig. 3d–f). Along the Oleander Line temperature at-depth anomalies (Fig. 4a–h) likely contributed to the South Slope MHWs, although the subsurface anomalies wouldn’t have been detected by the MHW detection algorithm.

For the winter 2015/16, the positive temperature anomalies along the Oleander line persisted at depth beyond the duration of the surface-defined MHWs. The subsurface temperature sections show almost uniform warming with depth over the shelf in November–December 2015. In Fig. 4f–g, the March–May 2016 period exhibits strong warming, exceeding 2–3 °C at depth and across the continental shelf.

#### Spring 2016

Beginning around May 2016, all regions saw an increase in the temperature anomaly. The jet stream position in April (Fig. 3g) was anomalously far north over almost the whole region, with a small trough at about 70° W. Additionally, the Gulf Stream appeared to be northwestward-shifted in April (Figs. 3g, 4g) as surface and at-depth temperature anomalies > 3 °C increased temperatures in the South Slope (Fig. 4f–h). The rest of the summer did not see an anomalously northward jet stream, although there was some variation in the path, nothing greater than 4°N of its usual position. The Center Shelf experienced a series of short, “moderate” MHWs starting in June and lasting until November (Fig. 3i–o). The South Slope entered a “moderate” MHW that lasted 41 days from late July to September. Surface-concentrated anomalies of > 1 °C can be seen during the same time period along the Oleander Line (Fig. 4i–l).

#### Summer and Fall 2016

In July 2016, SSTs began to increase across all of the Shelf/Slope sub-regions (Fig. 2). However, it was not until August when all regions, except for the North Shelf, started to experience MHW states. As time progressed, the warming became more uniform across the regions (Fig. 3k–o). The Gulf Stream’s path became “wigglier” in August (Fig. 3k–m) and strong warming signals were associated with the large meanders. Those “hot spots'' were concentrated in the slope regions from August to November 2016. The temperature anomalies at the Oleander Line started to deepen in September 2016 (Fig. 4l). In October 2016, the temperature anomalies extended throughout the water column (Fig. 4m). Then in November and December 2016 the anomalies at the Oleander Line decreased, but persisted subsurface (Fig. 4n–o).

SST anomalies peaked in October 2016 for the center and southern regions with anomalies between 1.4 and 2.1 °C. For the northern regions, SST anomalies peaked in November 2016 at 1.6–2.3 °C. Although other regions weren’t experiencing as strong SST anomalies, the uniform warming in November 2016 (Fig. 3n) is similar to the pattern in March for the 2012 MHW, in which an unusually northward-shifted jet stream suppressed heat loss from the ocean^4,8^. As previously mentioned, all regions experienced varying intensities of MHWs in November to December 2016. The Center Slope had a “moderate”, 26-day long MHW during this period. The North Shelf experienced a “strong” MHW that lasted 34 days in the same period. The North Slope experienced a 50-day long “strong” MHW. The jet stream was northward shifted over about 72–66° W in October to November (Fig. 3m,n).

The SST anomalies in late fall 2016 MHW differed from those in winter 2015/16 in their spatial expression. The winter 2015/16 exhibited strong warming in the center and southern regions, where the influence of the Gulf Stream impacted shelf and slope temperatures, while the northern regions remained cool (Fig. 3a–i). This contrasts with November 2016 when warming was relatively uniform across the Shelf/Slope regions and most regions entered a “moderate” or “strong” MHW state.

### The relative role of possible MHW drivers

**Figure 6:**
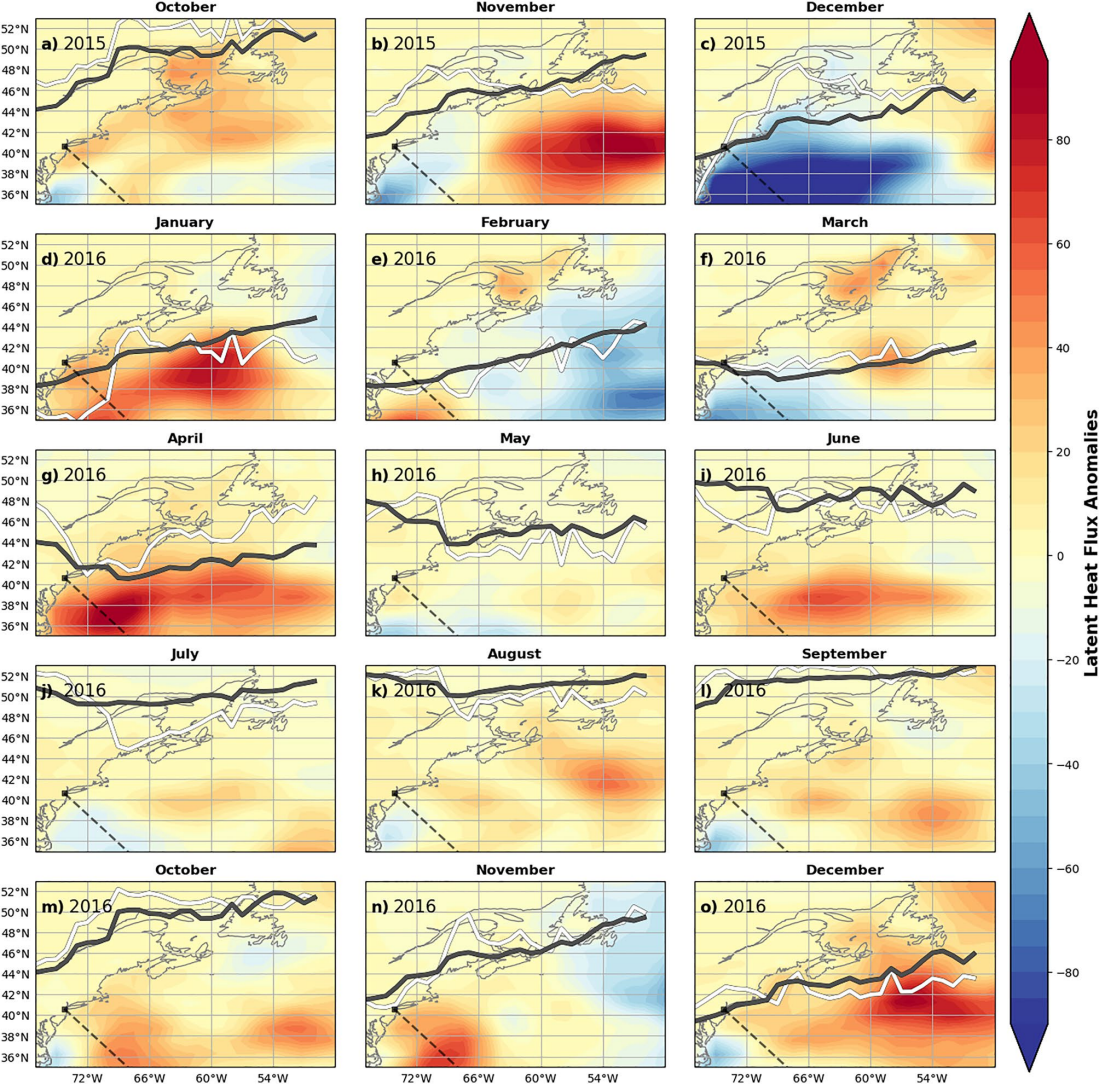
Latent heat flux anomaly for the 2015/16 period. Negative (positive) anomalies indicate the ocean is gaining (losing) more heat than usual for the 1986–2016 period. The thick gray line is the climatological position of the jet stream (1986–2016). The thick white line is the jet stream’s latitudinal position for 2015/16. These maps were generated using Python version 2.7.5.

Starting in late fall 2015, the jet stream remained north of its climatological position through the rest of 2015 (Fig. 6a–c), likely driving an increased heat flux south of the front from the warm subtropical air into the ocean. Usually the movement of the front in the fall brings cool polar air southward with it. This can be seen in Fig. 6c in which the center and southern regions have a negative heat flux anomaly, meaning that the region gained more heat than usual in December 2015.

It is likely that the increased heat flux into the ocean during December 2015 initiated the winter 2015/16 MHWs that the center and southern regions experienced. This is consistent with Schlegel et al.^25^, who found that latent heat flux is the most common driver of the start of MHWs in the NWA. The unusually active Gulf Stream meandering and WCR formations/absorptions in winter 2015/16 then further fueled the anomalous SST patterns observed in the Center and South Slope, as well as the anomalies seen at depth along the Oleander line from January to April 2016 (Fig. 4d–g).

The jet stream’s position throughout the rest of winter 2015/16 was consistent with its mean climatological position (Fig. 6d–f). This situation differs from 2012, when the jet stream had an anomalously northward-shifted position over the shelf regions during February and March (not shown). In 2016, there was no similar shift in March. Despite this difference in jet stream position in 2016 versus 2012, the 2016 SST anomalies were similar to 2012. This suggests other drivers must have maintained the high SST anomalies from January to April 2016 (Fig. 3d–g). Notably, strong positive latent heat flux anomalies are seen in November 2015, January 2016, April 2016, and December 2016 (Fig. 6b,d,g,o) indicates the ocean was losing more heat than usual. In addition to the negative latent heat flux anomalies seen in December 2015, negative anomalies are seen in center and northern regions in February 2016 (Fig. 6e), in southern regions in March 2016 (Fig. 6f), and northern regions in November 2016 (Fig. 6n). For the remaining months of 2016, May to October 2016, latent heat fluxes are not too anomalously positive or negative (Fig. 6h–m).

In late fall 2016, the jet stream shifted northward into an anomalous position over about 72–66° W in October to November 2016 (Fig. 6m,n). Combined with a uniform “strong” MHW event across the northern regions and negative latent heat flux over the same region (Fig. 6m,n) points to an atmospherically-forced MHW for the late fall 2016.

## Discussion

Our analysis suggests that multiple processes played a role in driving the MHWs during 2015/16. The fall 2015 MHWs seemed to be mostly atmospherically-driven and coincided with an anomalously northward-shifted and persistent jet stream, which has previously been shown^4,8^ to influence the shelf temperatures of the NWA. The jet stream position in December 2015 heatwave seemed to initiate a winter 2015/16 MHW. SSTs were already anomalously warm, although not technically in a MHW state. With SST anomalies that were well-above the mean leading into the winter, the winter 2015/16 MHW was then likely maintained by ocean advection. The pre-conditioning from the fall likely increased the intensity of the winter MHWs. During the winter 2015/16 large amplitude meanders of the Gulf Stream appeared south of Nova Scotia and the Gulf of St. Lawrence, resulting in very large temperature anomalies over the continental slope. More WCRs than usual (11 versus 3) were absorbed into the Slope from January to March 2016 (Fig. 5) and likely fueled a “moderate'' MHW in the Center Shelf and “strong” MHWs in the Center Slope, South Shelf, and South Slope. The MHW during the fall of 2016 was also likely forced by the jet stream remaining well north of its normal position. The warm conditions in the fall occurred over a wide shelf region in the Middle Atlantic Bight, especially in October. This pattern is consistent with atmospheric forcing of the MHW, similar to the 2012 event. In summary, for these MHW events, an interplay of atmospheric and oceanic drivers initiated and maintained MHWs in the NWA during 2015/16.

Globally, the intensity and number of MHW events is projected to increase^1^. Although much focus is on the surface expression of the MHWs, there are extreme warming events in the subsurface water masses as well, as corroborated by the Oleander temperature sections examined here, and these subsurface expressions can have wide-reaching impacts on marine ecosystems. Gawarkiewicz et al.^14,32^ have shown, using recent observations from the Pioneer Array, that intrusions of warm, salty water associated with WCRs that impinge on the upper-slope and outer shelf are now resulting in intrusions that reach much further onshore across the continental shelf than in previous decades. This may have significant impacts on both the region’s primary productivity and fishing outcomes during WCR intrusions. Gawarkiewicz et al.^14^ noted that annual average chlorophyll-a distributions during times of on-shelf intrusion were the lowest shelf-wide concentrations reported for data going back to 1998. Thus, these warm subsurface features, which may occupy the shelf for up to 4 months, may have effects that extend throughout the year.

Additionally, Neto et al.^15^ and Brickman et al.^16^ have discussed the implications that a northward-shifted Gulf Stream interacting with the Labrador Current can have on long-term warming on the continental shelf. We acknowledge that this mechanism may have also impacted Shelf/Slope temperatures during the study period, but due to the lack of high-resolution sub-surface measurements at present we did not explore this for the whole NWA. A dedicated observational program or modelling study would be needed to investigate this mechanism as a driver for MHWs in the future.

Superimposed on the individual MHWs examined here, the average SST across the entire NWA in 2016 was higher than the climatological mean for the whole year, and was not just elevated during the MHW events. The persistent warmer-than-average temperatures in early 2012 caused lobster to molt in April, rather than the typical timing in July^5^. The annual cycle of lobster landings for 2016 was almost identical to that in 2012, presumably brought on by the warmer-than-average temperatures during the winter of 2015/16. However, as discussed in Pershing et al.^5^, markets were prepared for this early molt based on their prior experience in 2012 and thus prices did not crash as they had in 2012.

Though generally viewed as detrimental to economically important species like lobster^3,5,33^, elevated water temperatures are associated with increased presence of other mid-Atlantic species like blue crabs and longfin squid (Loligo) in northern regions like the Gulf of Maine^3,5^. The annual catch of Loligo skyrocketed in 2016. The average landings for the previous 9 years (2007–2015) was 10.8 hundred metric tonnes. The 2016 landings were 18.3 hundred metric tonnes, a ~ 70% increase from the previous 9 years. The 2016 catch was the largest since 1999^17^. Not only are persistently warmer conditions advantageous to some species and harmful to others^18^, but it is also possible that the timing of MHW events plays an important role in their ecological and economic impacts. Though further work is necessary to understand how the ecosystem responds to MHWs forced by different drivers, these extreme events may have substantial impact on lobster and squid, two commercially important species of New England.

## Data Availability

Use of the following datasets is gratefully acknowledged: NOAA High Resolution SST data v2.1 provided by the NOAA/OAR/ESRL PSL, Boulder, Colorado, USA, from their website at https://psl.noaa.gov/data/gridded/data.noaa.oisst.v2.highres.html, the XBT data product, gridded and monthly averaged from 1977 to 2018, is available at https://doi.org/10.5281/zenodo.3967332, the raw *CMV* Oleander data is available at http://oleander.bios.edu/data/. The Warm Core Ring Census for 1980–2017 is available from Biological and Chemical Oceanography Data Management office (BCO–DMO) at https://doi.org/10.26008/1912/bco-dmo.810182.1, the ECMWF Reanalysis (ERA5) data is available at https://cds.climate.copernicus.eu/cdsapp#!/home. The Jet Stream Visualization tool, jsviz, was developed by Sara Haines and is available at http://doi.org/10.5281/zenodo.4570931. The MHW detection algorithm was written by Eric C. J. Oliver and available at https://github.com/ecjoliver/marineHeatWaves. Files used by the author to calculate anomalies and plot figures are available upon request.
